# Confounding by indication affects antimicrobial risk factors for methicillin-resistant *Staphylococcus aureus* but not vancomycin-resistant enterococci acquisition

**DOI:** 10.1186/2047-2994-3-19

**Published:** 2014-06-01

**Authors:** Rupak Datta, Ken Kleinman, Sheryl Rifas-Shiman, Hilary Placzek, Julie Lankiewicz, Richard Platt, Susan S Huang

**Affiliations:** 1 University of California Irvine School of Medicine, Health Policy Research Institute, 100 Theory, Ste. 110, Irvine, CA 92697, California; 2 Department of Population Medicine, Harvard Medical School and Harvard Pilgrim Health Care Institute, 133 Brookline Avenue, 6th Floor, Boston, MA 02215, Massachusetts

**Keywords:** Antimicrobial predictors, MRSA, VRE, Confounding by indication

## Abstract

**Background:**

Observational studies rarely account for confounding by indication, whereby empiric antibiotics initiated for signs and symptoms of infection prior to the diagnosis of infection are then viewed as risk factors for infection. We evaluated whether confounding by indication impacts antimicrobial risk factors for methicillin-resistant *Staphylococcus aureus* (MRSA) and vancomycin-resistant enterococci (VRE) acquisition.

**Findings:**

We previously reported several predictors of MRSA and VRE acquisition in 967 intensive care unit (ICU) patients with no prior history of MRSA or VRE who had an initial negative screening culture followed by either a subsequent negative screening culture (controls) or positive screening or clinical culture (cases). Within and prior to this acquisition interval, we collected demographic, comorbidity, daily device and antibiotic utilization data. We now re-evaluate all antibiotics by medical record review for evidence of treatment for signs and symptoms ultimately attributable to MRSA or VRE. Generalized linear mixed models are used to assess variables associated with MRSA or VRE acquisition, accounting for clustering by ward. We find that exclusion of empiric antibiotics given for suspected infection affects 17% (113/661) of antibiotic prescriptions in 25% (60/244) of MRSA-positive patients but only 1% (5/491) of antibiotic prescriptions in 1% (3/227) of VRE-positive patients. In multivariate testing, fluoroquinolones are no longer associated with MRSA acquisition, and aminoglycosides are significantly protective (OR = 0.3, CI:0.1-0.7).

**Conclusions:**

Neglecting treatment indication may cause common empiric antibiotics to appear spuriously associated with MRSA acquisition. This effect is absent for VRE, likely because empiric therapy is infrequent given the low prevalence of VRE.

## Findings

### Background

MRSA and VRE are important causes of hospital morbidity and mortality [1,2]. Colonization with either pathogen confers substantial risks of subsequent infection. Within 18 months after acquisition, up to 33% of MRSA carriers and 8% of VRE carriers develop invasive disease, with post-discharge infections often requiring readmission [3,4]. These high risks of infection have fueled efforts to identify modifiable risk factors for acquisition. Several risk factors for MRSA and VRE acquisition are well-known including prolonged hospitalization, invasive devices, surgery, and environmental contamination [5-8]. Other modifiable risk factors commonly include recent treatment with antibiotics. Numerous case–control studies, including our own, have shown a significant association between pathogen acquisition and fluoroquinolones, cephalosporins, and other antibiotics [9-13].

However, none of these studies accounted for confounding by indication [9-13], an unrecognized yet common limitation in case–control studies. Confounding by indication occurs when the effects of treatment indication are ignored, or in general, when factors that may be a consequence of a condition are instead treated as potential causes of that condition [14,15]. In this setting, confounding by indication may occur when empiric antibiotics are prescribed for signs and symptoms of an MRSA or VRE infection prior to the diagnosis of infection, and then the same antibiotics are viewed as risk factors for the infection. In such cases antibiotics represent a consequence of infection, rather than a potential cause.

Studies evaluating antibiotic exposures prior to culture confirmation of MRSA or VRE may falsely assume that antibiotics predict acquisition when, in actuality, they may have been given empirically for infectious symptoms related to MRSA or VRE. For example, pneumonia in an ICU patient with no prior history of MRSA may be treated with levofloxacin and vancomycin. If clinical cultures ordered days later demonstrate MRSA, assessments may spuriously conclude that both antibiotics are associated with acquisition since treatment preceded the MRSA-positive culture dates. In this study, we sought to assess whether the exclusion of antibiotics initiated for suspected infection ultimately attributed to MRSA or VRE changed antimicrobial risk factors for acquisition.

## Methods

We previously reported several factors, including antibiotics, associated with MRSA and VRE acquisition in a case–control study of 967 ICU patients from a 750-bed tertiary care center in Boston, Massachusetts between September 2003 and April 2005 [12]. For this article, we re-examined antibiotic exposures and determined whether antibiotics were initiated for signs and symptoms of an infection later attributed to MRSA or VRE. This study was approved by the Institutional Review Board at Brigham and Women’s Hospital.

Study procedures were described previously [12]. Briefly, we identified all patients with no prior history of MRSA who had an initial negative screening culture followed by either a subsequent negative screening culture (controls) or a positive screening or clinical culture (cases). From this cohort, we selected all cases and a random sample of controls for MRSA and a separate random sample of controls for VRE cases at a 1:1 ratio. There was no minimal ICU time requirement for cases and controls, and screening reflected high-compliance admission and weekly surveillance nares cultures for MRSA and rectal cultures for VRE. Within and prior to this eligible interval for acquisition, we collected multiple variables including diabetes, end-stage renal disease, chronic liver disease, solid and hematologic malignancies, time from ICU admission to initial negative screening culture, wounds, rashes, surgery, intubation, bronchoscopy, central lines, drains, tubes, albumin and creatinine levels, colonization pressure, and the presence of antibiotic-susceptible strains. Colonization pressure of MRSA (or VRE) was defined as the sum of daily number of same ward MRSA-positive (or VRE-positive) patients to which patients were exposed during the eligible interval for acquisition and categorized as follows: 0, 1- < 4, 4- < 8, 8- < 12, and ≥12. Time from ICU admission to initial negative screening swab was also categorized (1 day, 2 days, and ≥ 3 days).

All antibiotics were re-evaluated by medical record review for evidence of treatment for symptoms ultimately attributable to MRSA or VRE. Antibiotic administration was assessed for the time period encompassing two weeks prior to the initial negative surveillance culture until the time of subsequent negative or positive surveillance or clinical culture. Antibiotics were classified as follows: narrow-spectrum penicillins, broad-spectrum penicillins; first, second and third-generation cephalosporins; fluoroquinolones; carbapenems; aminoglycosides; macrolides; anti-MRSA (vancomycin, linezolid, synercid, daptomycin, tigecycline); and anti-VRE (linezolid, synercid, daptomycin, tigecycline) antibiotics. All antibiotics initiated empirically for signs and symptoms later attributed to MRSA or VRE infection were reviewed and confirmed by an infectious diseases physician. Antibiotics given for co-infection with another pathogen were retained.

### Statistical methods

The association between antibiotics and acquisition was first assessed using bivariate models. Variables with p < 0.1 were entered into multivariable logistic regression models. Final models were constructed by retaining variables with p < 0.05 in the multivariable models [12]. Bivariate and multivariable assessments accounted for clustering by ICU ward using generalized linear mixed models logistic regression. All analyses were conducted using SAS 9.3 (SAS Institute, Cary, NC).

## Results

We identified 244 cases and 248 controls for MRSA and 227 cases and 248 controls for VRE during the study period. Patient characteristics have been summarized previously [12]. Briefly, MRSA and VRE groups were similar, with 52% > 65 years-old, 25% with solid cancers, and 82% undergoing surgery.

Exclusion of empiric antibiotics given for signs and symptoms ultimately due to infection affected 17% (113/661) of antibiotic prescriptions among MRSA-positive patients but only 1% (5/491) of antibiotic prescriptions among VRE-positive patients. The most commonly affected antibiotics among MRSA-positive patients included anti-MRSA antibiotics (41 exclusions of 182 prescriptions), fluoroquinolones (30 exclusions of 193 prescriptions), third-generation cephalosporins (25 exclusions of 63 prescriptions), aminoglycosides (6 exclusions of 24 prescriptions), and clindamycin (4 exclusions of 18 prescriptions). No second-generation cephalosporins or broad-spectrum penicillins were prescribed for symptoms later attributed to MRSA infections. In the VRE group, few patients experienced infection, and thus very few antibiotics were attributable to signs or symptoms of infection. Only 1 aminoglycoside, 1 fluoroquinolone, and 3 third-generation cephalosporin prescriptions were excluded. Overall, accounting for treatment indication impacted 25% (60/244) of MRSA-positive patients and 1% (3/227) of VRE-positive patients.

Exclusion of antibiotics initiated empirically for signs and symptoms ultimately due to MRSA affected risk factors for acquisition (Table 1). Fluoroquinolones were no longer associated with acquisition, and aminoglycosides were found to be significantly protective (OR = 0.3, CI:0.1-0.7). This effect persisted when forcing fluoroquinolone prescriptions into the model excluding antibiotics initiated empirically for suspected MRSA infection (fluoroquinolones OR = 1.1, CI:0.7-1.7; aminoglycosides OR = 0.3, CI:0.1-0.7). Risk factors for VRE acquisition were not reevaluated due to the minimal impact of antibiotic exclusion.

**Table 1:** **Variables associated with methicillin-resistant *Staphylococcus aureus *acquisition before and after accounting for confounding by indication**

**Variable**	**Odds ratio (95% CI), *P *Value**	
	**Before accounting for confounding by indication**^**a**^	**After accounting for confounding by indication**
Methicillin-sensitive *S. aureus* carrier	0.5 (0.3, 1.0), 0.03	0.4 (0.2, 0.8), 0.01
Intubation	4.7 (1.8, 12.3), 0.002	5.3 (2.0, 14.4), 0.001
Fluoroquinolone	1.9 (1.2, 3.0), 0.01	-
Aminoglycoside	-	0.3 (0.1, 0.7), 0.003
Days from ICU admission to negative swab	< .0001	< .0001
1	1.0, reference	1.0, reference
2	2.0 (1.2, 3.3)	2.2 (1.3, 3.7)
≥ 3	15.6 (8.4, 29.0)	19.6 (10.4, 37.1)
Chronic liver disease	-	4.1 (1.2, 13.6), 0.02

^a^Previously reported elsewhere [12].

## Discussion

When identifying predictors of MRSA acquisition, failure to account for treatment indication caused common empiric antibiotics to appear falsely associated with MRSA acquisition. Such confounding has not been addressed in published case–control studies [9-13] and may occur when empiric antibiotics pre-date MRSA cultures that define acquisition. In future studies, investigators should address the possibility of confounding by indication in data analysis and interpretation.

Several hospital studies report that MRSA carriage is associated with fluoroquinolones [9,11-13,16], an empiric agent commonly prescribed for infections requiring hospitalization. However, we note that this artifactual association frequently occurs with other empiric antibiotics. In our data, approximately 25% of prescriptions for fluoroquinolones, third-generation cephalosporins, aminoglycosides and anti-MRSA antibiotics were excluded after accounting for treatment indication. These findings are consistent with the use of fluoroquinolones as a common first-line agent for empiric therapy at our institution during the study period. After excluding empiric antibiotics used for suspected MRSA infection, fluoroquinolones were no longer associated with MRSA. In addition, aminoglycosides were found to be significantly protective of acquisition, a plausible finding given its activity against many MRSA isolates [17].

In contrast to MRSA, confounding by indication was infrequent for VRE. This finding is likely due to the low prevalence of VRE infection in our ICU population despite oncology and transplant services in our hospital [2,4]. Thus, accounting for treatment indication had no impact on antimicrobial risk factors for VRE.

This work has important limitations. First, this ICU study from a large tertiary care center may lack generalizability. Second, these data were often reliant on single-site screening cultures to determine negative carriage status. However, all patients had no prior history of carriage based on microbiology records dating back to 1987 [12]. Third, extranasal MRSA carriage and multiple VRE rectal screens were not assessed to corroborate acquisition. Thus, results may not apply to clones that colonize non-surveillance sites.

In conclusion, we show that neglecting treatment indication may cause common empiric antibiotics to appear falsely associated with MRSA acquisition. These findings are relevant to the common practice of conducting retrospective cohort and case–control studies to determine antimicrobial risk factors for acquisition of multi-drug resistant organisms. Commonly used empiric agents such as fluoroquinolones may spuriously appear to increase the risk of pathogen acquisition if treatment indication is ignored. Readers should use caution in interpreting assessments of antibiotic exposures for conditions often treated with antibiotics.

## Abbreviations

MRSA: Methicillin-resistant *Staphylococcus aureus*; VRE: Vancomycin-resistant enterococci; ICU: Intensive care unit; OR: Odds ratio; CI: 95% confidence interval.

## Competing interests

All authors declare that they have no competing interests.

## Authors’ contributions

JL participated in the study design and coordination. RD and HP performed the data collection and contributed to the study design. SRS and KK participated in the study conception and design and performed the statistical analysis. RD and SH contributed to the data interpretation and manuscript preparation. SH and RP conceived of the study, contributed to its design and analysis, and critically revised the manuscript. All authors read and approved the final manuscript.
